# Soybean Salt Tolerance 1 (*GmST1*) Reduces ROS Production, Enhances ABA Sensitivity, and Abiotic Stress Tolerance in *Arabidopsis thaliana*

**DOI:** 10.3389/fpls.2016.00445

**Published:** 2016-04-11

**Authors:** Shuxin Ren, Chimera Lyle, Guo-liang Jiang, Abhishek Penumala

**Affiliations:** ^1^Agricultural Research Station, Virginia State UniversityPetersburg, VA, USA; ^2^Mills E. Godwin High SchoolHenrico, VA, USA

**Keywords:** soybean, *GmST1* overexpression, salt tolerance, drought tolerance, ROS production, ABA sensitivity

## Abstract

Abiotic stresses, including high soil salinity, significantly reduce crop production worldwide. Salt tolerance in plants is a complex trait and is regulated by multiple mechanisms. Understanding the mechanisms and dissecting the components on their regulatory pathways will provide new insights, leading to novel strategies for the improvement of salt tolerance in agricultural and economic crops of importance. Here we report that soybean salt tolerance 1, named *GmST1*, exhibited strong tolerance to salt stress in the *Arabidopsis* transgenic lines. The *GmST1*-overexpressed *Arabidopsis* also increased sensitivity to ABA and decreased production of reactive oxygen species under salt stress. In addition, *GmST1* significantly improved drought tolerance in *Arabidopsis* transgenic lines. *GmST1* belongs to a 3-prime part of Glyma.03g171600 gene in the current version of soybean genome sequence annotation. However, comparative reverse transcription-polymerase chain reaction analysis around Glyma.03g171600 genomic region confirmed that *GmST1* might serve as an intact gene in soybean leaf tissues. Unlike Glyma.03g171600 which was not expressed in leaves, *GmST1* was strongly induced by salt treatment in the leaf tissues. By promoter analysis, a TATA box was detected to be positioned close to *GmST1* start codon and a putative ABRE and a DRE *cis*-acting elements were identified at about 1 kb upstream of *GmST1* gene. The data also indicated that *GmST1*-transgenic lines survived under drought stress and showed a significantly lower water loss than non-transgenic lines. In summary, our results suggest that overexpression of *GmST1* significantly improves *Arabidopsis* tolerance to both salt and drought stresses and the gene may be a potential candidate for genetic engineering of salt- and drought-tolerant crops.

## Introduction

Soil salinity is one of the major environmental factors that significantly affected crop productivity and quality (Allakhverdiev et al., 2000). Large number of arable lands are being removed from crop production due to increased soil salinity (Epstein et al., 1980), and over 25% of the world’s potential arable land is currently contaminated by salt in different ways including irrigation, overuse of fertilizers and/or seawater intrusion (Pathan et al., 2007). Decreasing of the acreage of arable land for crop production has become a severe threat to global food security as more food will be needed to feed the growing population.

Soybean is considered a salt-sensitive glycophyte, with all developmental stages adversely affected by salinity stress (Phang et al., 2008). High salt levels generate a two-component stress on plants: an osmotic stress caused by reduced water availability in soil and an ionic stress due to imbalance of solutes in the cytosol (Blumwald et al., 2000; Conde et al., 2011). During soybean development, salt stress significantly reduces plant height and leaf size (Wang et al., 2001; Essa, 2002), decreases the number of internodes and pods (Phang et al., 2008), decreases protein content and seed quality, and causes a reduction in chlorophyll content (Lu et al., 2009). Salt stress also significantly affects seed germination, seedling growth, biomass, and seed yield (Abel and MacKenzie, 1964; Katerji et al., 1998; Wang and Shannon, 1999; Essa, 2002). The increase in salt content in soil could cause a concomitant decrease of up to 40% in soybean yield (Chang et al., 1994). Development of more precise salt tolerant cultivars will help reduce the detrimental loss of yield in soybean production in the areas with elevated salt levels.

Mechanisms of plant salt tolerance have been extensively studied and well characterized in many species (Lauchli, 1984; Zhu, 2002; Lenis et al., 2011). Both ABA-dependent and –independent pathways have been characterized, including identification of many key genes (Zhu, 2002). In addition, the role of calcium as a key secondary messenger in plant salt tolerance is well established in *Arabidopsis* (Mahajan et al., 2008). Soybean plants have developed several mechanisms to cope with salt stress conferring a wide spectrum of salt tolerance among genotypes (Phang et al., 2008). Mechanisms of salt tolerance include maintaining ion homeostasis by withholding toxic ions from sensitive aerial parts, adjusting osmotic potential in cells by accumulating metabolites, and restoring oxidative balance to prevent further damage due to excess accumulation of reactive oxygen species (ROS) (Phang et al., 2008). Understanding the mechanisms and identifying the genes involved in salt stress tolerance of soybeans will enable breeders to develop new strategies to enhance salt tolerance.

Due to the complexity of the soybean genome, mechanisms conferring salt tolerance are often overlooked. Nevertheless, with advances in high throughput sequencing technology, the full soybean genome has recently been sequenced (Schmuta et al., 2010). The availability of the entire soybean genome sequence provides ample opportunities for basic research to reveal the underlying mechanisms of salt tolerance. Many putative components of the salt tolerance signaling network homologous to those identified in *Arabidopsis* have been elucidated in soybean using the reverse genetic approach. Of these, GmSCA1, GmCaMs, GmSTL, GmAAPK, GmSTY1, and GmCIPK1 are involved in calcium signaling and eventually regulated downstream transcription factors and the effector genes for salt stress responses (Chung et al., 2000; Park et al., 2004; Luo et al., 2005; Li et al., 2006; Xu et al., 2006). Several soybean DREB homologs identified as regulators on the ABA-independent pathway were also shown to play roles in the regulation of salt tolerance (Chen et al., 2007). In addition, the ABA-dependent pathway also plays important functions in regulating soybean salt tolerance. Several transcriptional factors induced by both ABA and salt stress were successfully identified in soybean, including GmTDF-5, GmbZIPs, WRKY transcriptional factor family members, GmAP2/ERFs, and GmNACs (NAC transcriptional regulators) (Aoki et al., 2005; Meng et al., 2007; Liao et al., 2008, 2010; Zhang et al., 2008; Zhou et al., 2008; Zhai et al., 2013). These genes have a similar function in *Arabidopsis*.

Ion transporter genes are also believed to play essential roles in salt tolerance. Many putative ion transporters identified in soybean have also demonstrated a relationship between gene expression and salt tolerance (Luo et al., 2005; Li et al., 2006; Sun et al., 2006). More recently, through integration of whole genome sequencing/transcriptome sequencing and map-based cloning approaches, Guan et al. (2014b) and Qi et al. (2014) independently identified Glyma03g32900 as a potential candidate gene of salt tolerance in both wild and cultivated soybeans. Glyma03g32900 encodes a putative cation proton antiporter, *GmCHX1*, and is homologous to *Arabidopsis* cation proton antiporter *AtCHX20* (Qi et al., 2014). However, neither report provided solid *in-planta* evidence demonstrating a strong salt tolerant phenotype as a result of overexpressing the gene. Moreover, the gene structure described by Qi et al. (2014) is different from what it was deposited in NCBI (http://www.ncbi.nlm.nih.gov/nuccore/kf879912), and the expression patterns observed by Guan et al. (2014b) are inconsistent with the role of this gene played in salt tolerance.

Previously, we characterized soybean salt tolerance in WF-7 and mapped a major QTL in the same region on linkage group N that was closely linked with a microsatellite marker, JD33–432 (Ren et al., 2009, 2012). Using the first draft of the soybean genome sequence, and through bioinformatics analysis, we identified two putative cation/proton antiporters on scaffold 63 nearby JD33–432. These two genes were, at the time, named Gm0063×00340 and Gm0063×00341. According to the update soybean sequence database available currently, these two genes are overlapped with Glyma03g32900 (Guan et al., 2014b; Qi et al., 2014) and are predicted as one gene, Glyma.03g171600 (Qi et al., 2014), in the current Wm82.a2 assembly. Here we present the evidence that overexpression of Gm0063×00340, renamed *GmST1*, produced strong tolerance to salt stress in *Arabidopsis* seedling and adult stages. In addition, the *GmST1*-transgenic *Arabidopsis* lines also increased sensitivity to ABA and decreased production of ROS under salt stress. Furthermore, the *Arabidopsis GmST1*-transgenic lines also improved tolerance to drought stress. Based on the current version of soybean genome sequence annotation, *GmST1* was located within Glyma03g171600, and Glyma03g171600 overlaps with Glyma03g32900. Thus we propose that at least two different models of gene prediction exist for this region. By comparative RT-PCR analysis, *GmST1* has been demonstrated as an intact gene functioning in soybean leaf tissues, not a part of Glyma03g171600.

## Materials and Methods

### Soybean Materials

Two soybean varieties, WF-7, salt tolerant (Ren et al., 2009, 2012), and Union, sensitive to salt stress, were used in this study. WF-7 was kindly provided by Dr. Lijuan Qiu, Chinese Academy of Agricultural Science, Beijing, China. Union was originally received from the USDA Soybean Germplasm Collection (PI548622) and was maintained at the Virginia State University Soybean Breeding Program. Both varieties were grown in a greenhouse with ambient temperature and natural light.

### *Arabidopsis* Transgenic Lines

To produce *Arabidopsis* transgenic lines, *Arabidopsis* ecotype Col-0 was used. The DNA fragment covering start and stop codons of *GmST1* was amplified from WF-7 genomic DNA using primers GmST1-F (P4) and GmST1-R (P6), cloned into TOPO vector PCR2.1 and sequence-confirmed. The confirmed DNA fragment was then inserted into binary vector pCBK05 between XbaI (5′ end) and SacI (3′ end) sites, driven by the CaMV 35S promoter. The plasmid containing 35S::GmST1 construct was transformed first into *Agrobacterium tumefaciens* GV3101 and then into *Arabidopsis* ecotype Col-0 by the floral dipping method (Clough and Bent, 1998). Two independent single-copy homozygous transgenic lines were generated through herbicide resistance selection. Briefly, primary transformants were identified and determined based on the presence of herbicide resistance that is completely linked with the target gene. Individual transgenic lines were further genetically analyzed using herbicide resistance as the indicator in the following T_1_ and T_2_ generations to identify single copy T-DNA insertion lines. Two independent homozygous transgenic lines were then bred and the seeds from T_3_ and/or T_4_ generations were subjected to various stress tests.

*Arabidopsis* wild type Col-0 and homozygous *GmST1*-transgenic lines were grown in soil in a growth chamber with 14/10 light-dark cycles at a light intensity of approximately 130 μmol m^-2^s^-1^, 21°C temperature and 60% relative humidity. For germination and seedling assays, *Arabidopsis* seeds were first surface-sterilized with 50% (v/v) bleach and 0.1% (v/v) Triton X-100 for 7.5 min, and were cold-treated at 4°C for 24 h. The cold-treated seeds were then grown on Murashige and Skoog (MS) solid media with 1% sucrose under continuous light of approximately 50 μmol m^-2^s^-1^. The seedlings/plants were maintained in the growth chamber under the same conditions until sampling for experiments as describe below.

### *GmST1* Overexpressing in *Arabidopsis* and Transgenic Line Confirmation

To determine the expression of *GmST1* in the transgenic *Arabidopsis* lines, total RNA was isolated from leaves of 3-week-old seedlings of wild type and two independent transgenic lines using TRI reagent (Sigma; St. Louis, MO, USA), following manufacture’s instruction. RT-PCR was conducted to examine the expression of *GmST1* in *Arabidopsis* transgenic lines using primer pair of P4 and P6. After reverse transcription step (Invitrogen Reverse Transcriptase III was used), 30 cycles of PCR amplification (95°C, 30 s-60°C, 30 s-72°C, 1 min) was performed. *Arabidopsis* actin 1 gene was served as a loading control.

### Seed Germination under ABA and NaCl Stress and Seedling Salt Tolerance Assay

To investigate the role and mechanism(s) of *GmST1* in abiotic stress tolerance, the wild type and transgenic lines were first subjected to seed germination tests in response to ABA and NaCl stresses. Surface-sterilized and cold-treated seeds were planted on MS medium supplemented with (1) 1, 2.5, and 5 μm ABA; and (2) 50 and 100 mM NaCl, with no ABA- and/or NaCl-supplemented treatment used as the control. All seeds were germinated under continuous light, and germination rate was recorded daily for 4 days. To test salt tolerance ability at seedling stage, 4-day-old germinated seeds of wild type and transgenic lines were transferred to fresh MS medium with or without 100 mM NaCl. All seedlings were grown vertically for additional 6 days under the same growing conditions. Photos were taken to visualize the phenotypes, and root lengths were measured. All tests were repeated twice, and Student’s *t*-test was used for statistical analysis.

### Detection of H_2_O_2_ Production

Salt stress-induced H_2_O_2_ production was detected using 3,3′-diaminobenzidine (DAB) staining as described by Xia et al. (2009). Four-week-old homozygous transgenic lines and wild type Col-0 were watered with 200 mM NaCl (treated) or water (untreated). After 24 h of treatment, two leaves from each individual plant were detached from salt treated and untreated plants, placed in 1 mg/ml DAB, and incubated at room temperature for 5 h. Samples were de-stained in boiling absolute ethanol, and then pictures were captured for record. Five plants from each transgenic line and wild type were used for H_2_O_2_ detection.

### Salt Tolerance Test at Adult Stage

Four-week-old wild type and transgenic lines grown in the growth chamber were tested for their salt tolerance ability. All pots containing tested lines were saturated with 200 mM NaCl for 6 days. Control pots were watered with the same amount of water. After the treatment, survival rates were recorded, and pictures were captured to show visible phenotypes. The experiment was repeated twice for statistical analysis.

### Water Loss Assay and Drought Tolerance Analysis

To examine the possible role of *GmST1* in drought stress tolerance, we first conducted water loss experiments using detached leaves. Three leaves were excised from each 4-week-old plant grown in the growth chamber, and the fresh weight was immediately determined. All leaves were placed on a laboratory bench at room temperature for 210 min. Every 30 min, leaf weight was recorded in the same order that leaves were detached from their corresponding plants. Water loss was calculated as the percentage of the initial fresh weight at each time point. For each genotype (Col-0, *GmST1–1*, and *GmST1–2*), five plants were tested for water loss.

To test drought tolerance at adult stage, wild type and a transgenic line were grown under normal watering conditions for 4 weeks and then subjected to drought stress by withdrawing irrigation. After 11 days of drought stress, drought tolerance phenotypes were examined, and pictures were captured to show the difference between the wild type and the transgenic line. A total of 15 plants for each of Col-0 and *GmST1–1* were evaluated for drought tolerance.

### RNA Extraction and RT-PCR Analysis

Given that different gene models might exist in the soybean genomic region where *GmST1* is located (Ren et al., 2009, 2012), we designed serial primers (**Table 1**) according to the different gene prediction models and examined *GmST1* structure in relation to other predicted models using RT-PCR analysis. WF-7 and Union seedlings at V3 stage were treated with 200 mM NaCl or H_2_O (control) for 48 h prior to RNA isolation. Total RNAs were extracted from the leaves using TRI^®^ reagent (Sigma; St. Louis, MO, USA) as per the manufacturer’s instructions. RNA was quantified using NanoDrop 2000 (Thermo Scientific; Wilmington, DE, USA), and its quality was monitored on 1.2% agarose gel. Five μg of total RNA for each sample was used for cDNA synthesis in a 20 μl reaction using SuperScript^®^ III (Invitrogen; Carlsbad, CA, USA) according to the manufacturer’s instructions, followed by PCR of 1 μl of the RT products using *Taq* DNA polymerase (New England BioLabs; Ipswich, MA, USA) with 35 cycles of amplification. The relative position of each designed primer in relation to the gene prediction models was illustrated in **Figure 7A**, and primers’ names and sequences were listed in **Table 1**.

**Table 1:** List of primers used in reverse transcription-polymerase chain reaction (RT-PCR) analysis of soybean and *Arabidopsis* lines.

Primer Name	Sequences
GmST1 F (P4)	5′-TCTAGAATGGCGTTTGTTGCAGCCATG -3′
GmST1 R(P6)	5′-GAGCTCTCATAAGGTTCGGGGATCCTTTC -3′
GmST1 Intron (P5)	5′-TTATTTAATTCAAGACTGTGCATC -3′
Gm0063×00341 F (P1)	5′-GGATCCATGACGTTCAACGCGAGCAC -3′
Gm0063×00341 R (P3)	5′-ACTAGTTTAAGTTATAACTATAGTAGGTCC -3′
Gm0063×00341 Exon2 (P2)	5′-TGGAATTTTGTTGGGGCCTTC-3′
GmTubulin1 F	5′-ATGAGAGAAATCTTGCACATCCAG-3′
GmTubulin1 R	5′-TAAGGCTCCACAACGGTATC3′-3′
AtActin1 F	5′-ATGCTGGTATCCATGAAACCACCT-3′
AtActin1 R	5′-CCTGTGAACAATCGATGGACCTGA-3′

### Analysis of Regulatory Elements on Putative Promoter of *GmST1*

To further define *GmST1* gene structure, the putative promoter, a 1,269 bp DNA fragment from the start codon of *GmST1* to the stop codon of the upstream gene (*Gm0063*×*00341*), was analyzed for its possible regulatory elements. TATA box was predicted using TSSP (Solovyev, 2001) and other regulatory elements were analyzed in NSite-PL software (Solovyev et al., 2010).

## Results

### Overexpressing Soybean Gene *GmST1* in *Arabidopsis*

**FIGURE 1:**
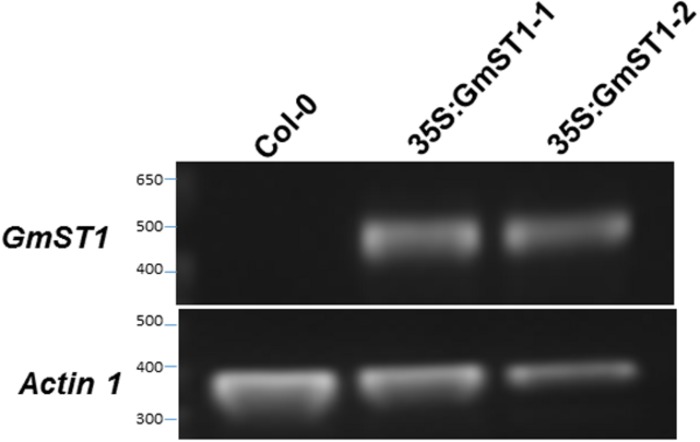
**Reverse transcription polymerase chain reaction (RT-PCR) confirmation of *GmST1* overexpression in *Arabidopsis thaliana***. Total RNA was isolated from leaves of wild type and two independent CAMV 35S:GmST1 *Arabidopsis* transgenic lines. RT-PCR was conducted using soybean primers P4 and P6. *Arabidopsis* actin 1 was used for loading control.

To examine the role of *GmST1* in salt tolerance in plants, we cloned a genomic DNA fragment covering the full length of cDNA of *GmST1* from soybean variety WF-7. Driven by CAMV 35S promoter, we transferred the construct into *Arabidopsis* ecotype Col-0. Transformants were selected based on herbicide resistance as the maker, and two independent homozygous transgenic lines carrying a single copy of T-DNA insertion were developed for further characterization of the roles of *GmST1* in abiotic stress tolerance. RT-PCR analysis showed high expression of *GmST1* in both *Arabidopsis* transgenic lines (**Figure 1**).

### *GmST1* Enhances Seed Germination and Root Elongation under Salt Stress

**FIGURE 2:**
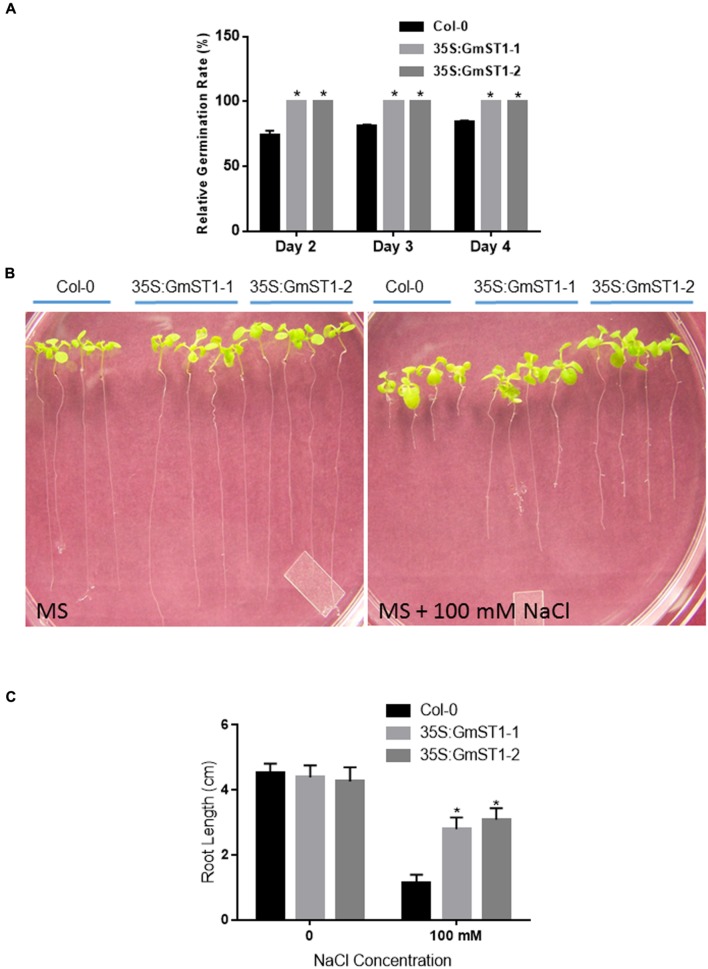
**Effect of GmST1 on *Arabidopsis* seed germination and seedling development under salt stress. (A)** Seed germination rate of *Arabidopsis* transgenic lines was enhanced by GmST1 under 100 mM NaCl condition. **(B)** Effect of GmST1 overexpression on *Arabidopsis* root development under control and 100 mM NaCl conditions. **(C)** Quantitative measurements of GmST1 effects on root growth. Experiments were biologically duplicated and bars represent means ± SD. ^∗^*P* < 0.05, as determined by Student’s *t*-test.

The transgenic lines were first investigated for their responses to salt stress during the germination and the seedling stages. Both transgenic lines, together with wild type Col-0, were germinated on MS medium supplemented with 50 mM or 100 mM NaCl. Comparing to MS control, there were no significant differences in seed germination between Col-0 and *GmST1* transgenic lines under 50 mM NaCl stress. All seeds were fast-germinated on day 2 (data not shown). However, under the stress treated with 100 mM NaCl, the seed germination of Col-0 was significantly inhibited, with only about 70% of seeds germinated on day 2 and 83% germinated by the end of experiment day 4 (**Figure 2A**). On the contrary, 100% of seeds were geminated at day 2 in both transgenic lines. These results demonstrated that *GmST1* expression improved seed germination under high salt stress.

Furthermore, we examined the effect of *GmST1* expression on root elongation under salt stress. Synchronized *Arabidopsis* seedlings with similar initial root length were vertically grown on MS medium with or without 100 mM NaCl. As shown in **Figures 2B,C**, on MS only medium, wild type and both *GmST1* transgenic lines grew normally with similar root elongation. However, under 100 mM NaCl stress, root elongation of Col-0 was significantly inhibited, with about 75% inhibition observed compared with the control without stress. On the other hand, both transgenic lines showed significant greater root elongation relative to that of Col-0 under the same salt condition. The root length of transgenic lines grew two times more than that of Col-0, and only about 25% inhibition was produced by salt stress compared with no salt stress applied. These results confirmed the role of *GmST1* in salt tolerance in responses both to seed germination and root elongation at seedling stage.

### *GmST1* Expression Confers Strong Salt Tolerance in *Arabidopsis* at Adult Stage

**FIGURE 3:**
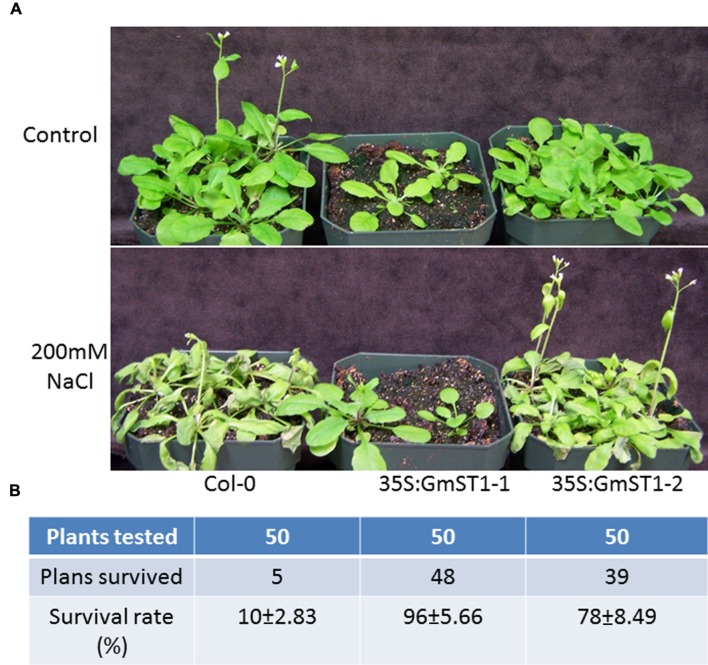
**Effect of GmST1 overexpression on *Arabidopsis* salt tolerance at adult stage. (A)** Overexpression of GmST1 improves salt tolerance ability in *Arabidopsis*. 200 mM NaCl were used to treat 3-week-old wild type Col-0, and two overexpressing lines (GmST1–1 and GmST1–2). Control plot was watered with tap water. **(B)** Survival rate of NaCl treated lines of Col-0, GmST1–1, and GmST1–2. Two independent experiments (with 25 plants each) were conducted.

Salt tolerance phenotype caused by *GmST1* at the seedling development stage prompted us to further test whether or not *GmST1* could improve salt tolerance at later stages during plants’ growth. As shown in the duplicated experiments with 4-week-old wild type Col-0 and transgenic lines (**Figures 3A,B**), all lines grew normally without NaCl treatment. The treatment with 200 mM NaCl caused severe damage on wild type Col-0, while no or less damage by NaCl was observed on either of the *GmST1* overexpression lines (**Figure 3A**). After 6 days of treatment, only 10% of wild type plants survived; however, 95 and 78% of survival rates were recorded for the two transgenic lines. These data strongly suggest that soybean *GmST1* expression would improve salt tolerance in *Arabidopsis*.

### *GmST1* Expression Reduces ROS Production during Salt Stress

**FIGURE 4:**
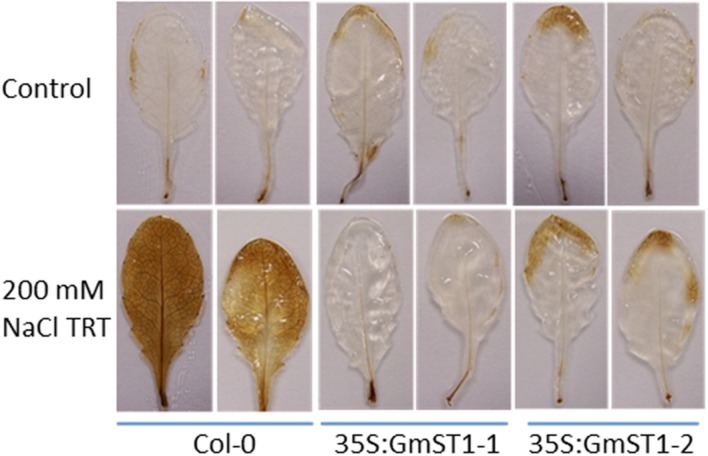
**Effect of GmST1 overexpression on reactive oxygen species (ROS) production under control and salt stress**. Hydrogen peroxide was detected by incubating leaves excised from *Arabidopsis* lines treated or untreated with 200 mM NaCl and stained by 1 mg/ml DAB solution for 5 h followed by de-staining. Upper panel represents *Arabidopsis* lines under control condition, and lower panel represents *Arabidopsis* lines treated with 200 mM NaCl.

Abiotic stresses cause damage to plants and lead to over production of ROS, such as H_2_O_2_ (Mustilli et al., 2002; Apel and Hirt, 2004). Regulation of ROS production is one of the mechanisms that crops use to defend against different biotic and abiotic stresses (Mustilli et al., 2002; Apel and Hirt, 2004; Jaspers and Kangasjarvi, 2010; Torres, 2010). To investigate if *GmST1*’s salt tolerance response involves ROS-related mechanism(s), we examined H_2_O_2_ production during salt stress. Leaves from both NaCl-treated and untreated control plants (4-week-old) of Col-0 and transgenic lines (five individual plants from each line/treatment) were excised and stained by DAB to detect the production of H_2_O_2_. As shown in **Figure 4**, there was no difference between Col-0 and transgenic lines in H_2_O_2_ production under the control condition (**Figure 4**, upper). However, when plants treated with 200 mM NaCl for 48 h, wild type Col-0 produced more H_2_O_2_ than that of either transgenic lines (**Figure 4**, lower), indicating a possible involvement of ROS signaling in GmST1-mediated salt tolerance.

### *GmST1* Enhances ABA Sensitivity in *Arabidopsis*

**FIGURE 5:**
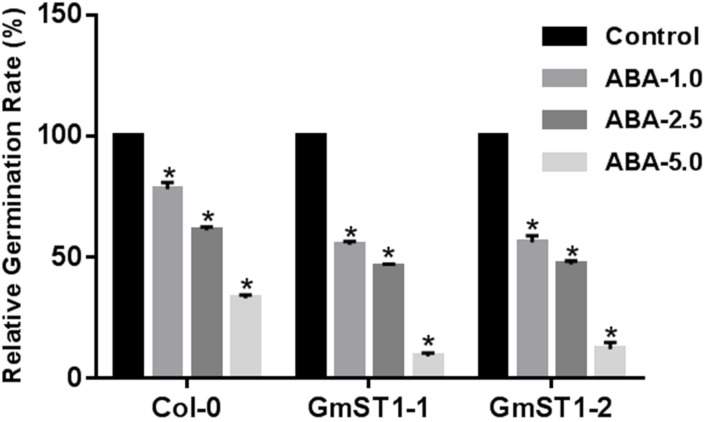
**Overexpression of GmST1 enhances ABA sensitivity**. Seeds of *Arabidopsis* lines (Col-0, GmST1–1, and GmST1–2) were germinated on MS medium supplemented with 0, 1.0 μM, 2.5 μM, and 5.0 μM ABA. Relative germination rate was calculated. Experiments were biologically duplicated. Bars represent means ± SD. ^∗^*P* < 0.05, as determined by Student’s *t*-test.

To further examine the role of *GmST1* in abiotic stress tolerance, we tested whether or not *GmST1* overexpression could alter ABA sensitivity during the seed germination stage. Seeds of Col-0 and two transgenic lines were surface-sterilized and germinated on MS media containing none or 1 μM, 2.5 μM, or 5 μM ABA. Shown in **Figure 5**, all seeds of either the transgenic lines or Col-0 were germinated on MS medium without ABA treatment. On the MS medium containing ABA, seed germination was inhibited by ABA in a dosage-dependent manner. Relatively, seed germination of the two transgenic lines were significantly more inhibited than that of Col-0 at all ABA concentration levels. At 5 μM ABA level, over 30% of Col-0 seeds germinated, while only about 10% seeds of transgenic lines were able to germinate. These results clearly demonstrate that *GmST1* regulates salt tolerance, at least in part, through an ABA-dependent pathway.

### *GmST1* Reduces Leaf Water Loss and Improves Drought Tolerance in *Arabidopsis*

**FIGURE 6:**
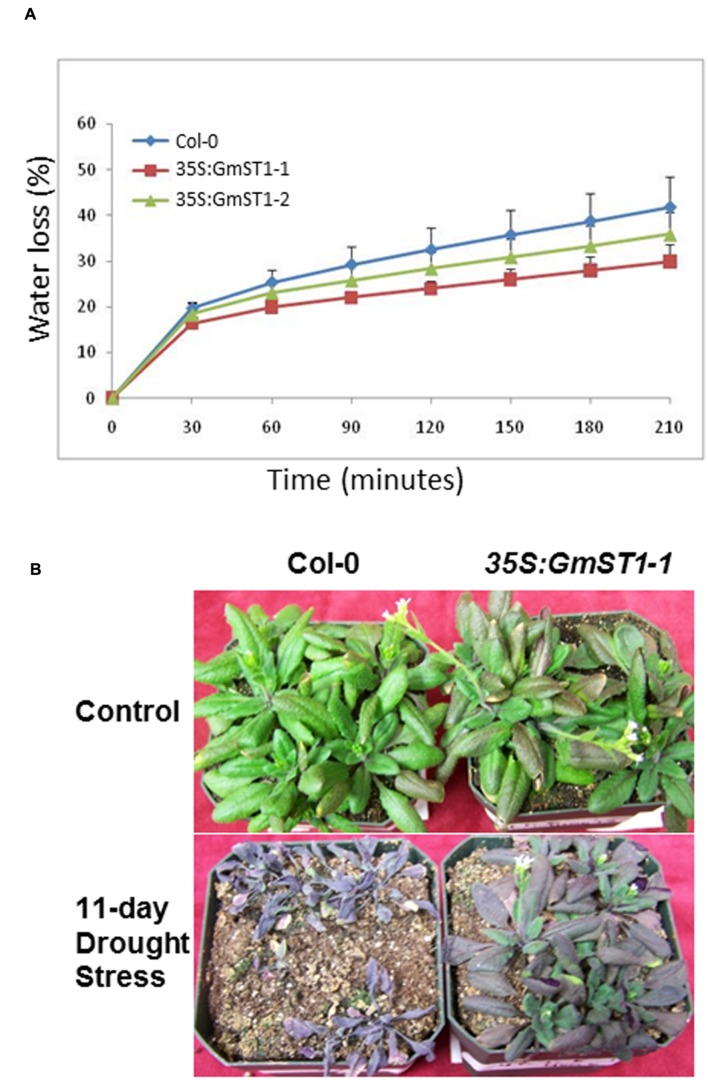
**Effect of GmST1 overexpression on *Arabidopsis* drought tolerance. (A)** Overexpression of GmST1 reduces water loss through transpiration. Leaves were excised from 4-week-old *Arabidopsis* lines (Col-0, GmST1–1, and GmST1–1) and monitored their water losses under ambient temperature for 210 min. Water loss was calculated as the percentage of initial fresh weight. **(B)** Overexpression of GmST1 confers drought tolerance in *Arabidopsis*. 4-week-old *Arabidopsis* plants (Col-0 and GmST1–1) were subjected to a water stress by withholding water. Photos were taken after 11 days of drought stress.

Since both ABA and ROS are regarded as classical signal pathways that plants evolved for defending against abiotic stresses, the fact that *GmST1* both increased ABA sensitivity and decreased ROS production under salt stress prompted us to further examine if it could also strengthen drought tolerance in *Arabidopsis*. We first conducted water loss experiments using detached leaves. As shown in **Figure 6A**, during a 210-min period, wild type Col-0 lost more than 40% of water due to evaporation, while two *GmST1* transgenic lines evaporated only less than 30% of water. The significant difference in water loss between Col-0 and the transgenic lines indicated a possible role of *GmST1* in plants’ drought tolerance. We further compared the drought tolerance between Col-0 and a transgenic line for an extended period of drought stress. After 11 days of water withholding, Col-0 plants died of stress (**Figure 6B**), while almost all the plants from the transgenic line survived. It suggests that *GmST1* may play an important role in drought stress tolerance in plants.

### *GmST1* Serves as an Intact Gene, Not Part of Glyma.03g171600, in Soybean Leaf Tissue

**FIGURE 7:**
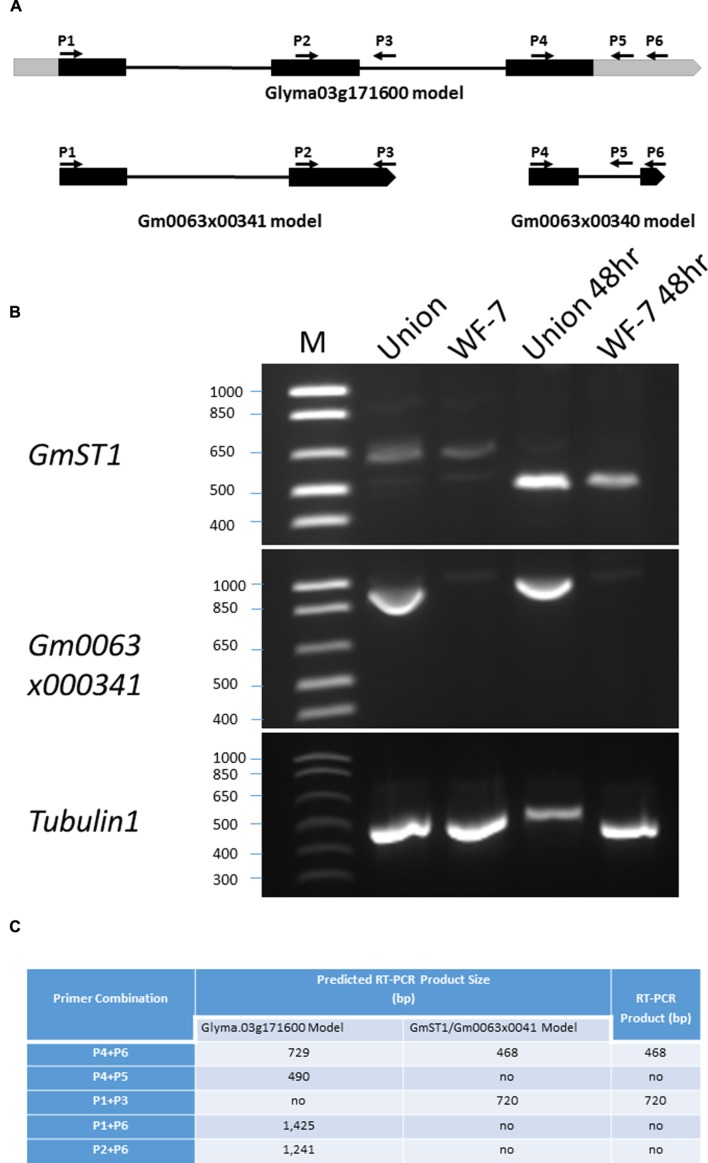
**Characterization of gene structure around *GmST1* genomic region. (A)** Schematic summary of different gene prediction models around *GmST1* genomic region. Black bars represent exons, gray bars are untranslated regions (5′-UTR and 3′-UTR), and lines represent introns in their expected gene models. Primer positions (P1 through P6) are indicated in response to different gene prediction models. **(B)** *GmST1* and Gm0063×00341 expression pattern analysis in soybean leaf tissues. RT-PCR was conducted using primer pairs corresponding to GmST1 and Gm0063×00341, respectively. Total RNA was isolated from the salt treated and untreated leaves of soybean varieties WF-7 and Union. **(C)** Prediction and experimental RT-PCR products around *GmST1* genomic region.

In the first draft of the soybean genome sequence, *GmST1* and Gm0063×00341 were initially annotated as two independent genes, both encoding putative cation/proton antiporters. In soybean genome sequence versions Glyma 1.0 and Glyma 1.1, this region was predicted to be a part of Glyma03g32900 (Schmuta et al., 2010). Later, in the newly released version Glyma 2.0, these two genes (*GmST1* and *Gm0063*×*00341*) were predicted to be a single gene, named Glyma.03g171600 (Qi et al., 2014), but with different intron-exon predictions. Given that different gene models were proposed in this region, we further characterized the *GmST1* gene structure in relation to Gm0063×00341 and Glyma.03g171600. The designated primers are listed in **Table 1** and their relative positions in the region are illustrated in **Figure 7A**.

In the *GmST1* model, the *GmST1* gene covers 729 bp genomic sequence with two exons and one intron. Its full length cDNA is 468 bp. In the Glyma.03g171600 model, the *GmST1* DNA fragment lies in its 4th exon with a 2 bp shift to the left and a 121 bp extra sequence at 3 prime end. However, no intron was predicted in this region in the Glyma.03g171600 model. Therefore, we first conducted RT-PCR analysis using Primer pair P4 and P6, which are *GmST1* forward and reverse primers covering the full length cDNA. As shown in **Figure 7B**, Primer combination P4 and P6 successfully amplified a strong RT-PCR product at about 500 bp in both salt-treated WF-7 and Union leaves, indicating that *GmST1* was induced by salt and that there was a predicted intron in *GmST1*. We then designed a reverse primer (P5) within the predicted intron region of *GmST1* but annotated as 3′ UTR in the Glyma.03g171600 model, and we performed RT-PCR using the P4 and P5 combination. If the Glyma.03g171600 model was correct for the case in our study, this combination should produce a 490 bp band. However, the RT-PCR analysis using P4 and P5 failed to amplify any PCR product (**Figure 7C**), indicating that P5 was located within the intron region.

In the Glyma.03g171600 model, Gm0063×00341 belongs to the 5′ part of the Glyma.03g171600 gene, and the primer P3 (**Figure 7A**), located at the 3′ end of Gm0063×00341, is predicted to be in the intron of the Glyma.03g171600 gene. We conducted RT-PCR using P1 and P3. If Gm0063×00341 was an intact gene, then the P1 and P3 combination should yield 720 bp RT-PCR product; and if the Glyma.03g171600 prediction model was correct, then it should not amplify any RT-PCR product. Shown in **Figure 7B**, we successfully amplified an RT-PCR product at about 720 bp in both salt-treated and untreated plants of the variety Union. These results clearly demonstrate that both *GmST1* and Gm0063×00341 are not part of the Glyma.03g171600 gene in soybean leaf tissue.

Furthermore, we discovered that the expression patterns of *GmST1* and Gm0063×00341 were significantly different. *GmST1* was induced by salt and strongly expressed in both WF-7 and Union upon salt treatment. On the other hand, Gm0063×00341 was highly expressed only in Union (both control and salt-treated samples) and its expression was not responsive to salt stress. To further examine whether or not *GmST1* and Gm0063×00341 belong to the same gene, we conducted RT-PCR analyses using primer pairs of P1 and P6, and P2 and P6 (where P2 is located on the second exon of the Gm0063×00341 gene). As predicted, neither primer combination yielded RT-PCR products at the corresponding sizes (**Figure 7C**). Taken together, our results strongly suggest that *GmST1* and Gm0063×00341 serve as independent genes in soybean leaf tissues.

### *GmST1* Promoter Analysis Reveals a Putative ABA Responsive Element and a Dehydration Responsive Element

To further confirm that *GmST1* served as an intact gene, we analyzed a 1,269 bp DNA fragment covering from the stop codon of the upstream gene and the start codon of GmST1. This fragment covers the whole sequence of the 2nd intron of Glyma.03g171600. Sequence analysis revealed 78% AT composition and a TATA box (TATAAA) at position -122. Furthermore, through NSite-PL prediction, we identified an ABRE element (GTCAAGTGTC) (Yamamoto et al., 2009) at position -985, and a DRE element (TTAGTCGGTT) (Chen H. et al., 2010) at position -1,049. The discovery of a putative functional promoter and possible stress-related *cis*-acting elements further indicate that *GmST1* may serve as an intact gene and play roles in soybean abiotic stress tolerance.

## Discussion

Abiotic stresses, including salinity, are a great challenge and major restricting factor for crop production worldwide. It was predicted that about 50% of the world arable land would be affected by salinity by 2050 (Blumwald and Grover, 2006). One of the best solutions to such agricultural challenge is to efficiently use genetic resources to develop salt tolerant cultivars. We previously characterized WF-7 as a salt-tolerant soybean variety (Ren et al., 2012) and mapped a major QTL associated with salt tolerance to the linkage group N near a microsatellite marker JD33–432 in the mapping population of WF-7 × Union (Ren et al., 2009). By initial bioinformatics search in the rough draft of the soybean genome sequence, a putative cation/proton antiporter was identified nearby JD33–432. In this study, we cloned this putative cation/proton antiporter, named soybean salt tolerance 1 (*GmST1*), and investigated its roles in abiotic stress tolerance when overexpressed in *Arabidopsis thaliana*. The results showed that overexpression of *GmST1* in *Arabidopsis* produced strong tolerance to salt stress at both seedling and adult stages (**Figures 2** and **3**). Moreover, given that *GmST1* caused transgenic *Arabidopsis* lines to exhibit more sensitivity to ABA (**Figure 5**), we speculate that *GmST1* may regulate salt tolerance through an ABA-dependent pathway. The *GmST1* encodes a putative cation/proton antiporter. Many cation/proton antiporters have been cloned from different plant species, and their potential roles in salt tolerance also have been clearly demonstrated (Shi et al., 2000; Kato et al., 2001; Brini et al., 2007). However, mechanisms in regulating salt tolerance by cation/proton antiporters still remain uncovered. For example, *Arabidopsis SOS1* and *AtHKT1* both conferred salt tolerance (Shi et al., 2000; Kato et al., 2001; Rus et al., 2001). Even though ABA-responsive elements were discovered in their promoter regions (Osakabe et al., 2014), *SOS1* seemed to be regulated by an ABA-independent pathway (Yamaguchi-Shinozaki and Shinozaki, 2006), while *AtHKT1* was regulated partially by ABA-dependent signaling pathway (Shkolnik-Inbar et al., 2013). We previously found that the salt tolerance in WF-7 was partially regulated by ABA (Ren et al., 2012). Although we did not directly test if *GmST1* was induced by ABA in soybean, an ABRE *cis*-acting element (Bonetta and McCourt, 1998; Yamamoto et al., 2009) and a DRE *cis*-acting element (Chen H. et al., 2010) were identified in its putative promoter region. Additionally, overexpression of *GmST1* in *Arabidopsis* enhanced ABA sensitivity. Taken together, these results assured at least a partial involvement of ABA in *GmST1*-mediated salt tolerance.

ABA is considered an abiotic stress hormone, and in most cases, an ABA-dependent signaling pathway regulates plant responses to multiple stresses (Larkindale and Knight, 2002; Zhu, 2002; Fujita et al., 2005; Chen L.T. et al., 2010). Thus, we further tested *GmST1*’s ability to defend against drought stress, the major cause of crop losses worldwide. The results indicated that *GmST1* expression prevented water loss from leaves and improved drought tolerance during the adult stage (**Figure 6**). This is not surprising, though, as many other cation/proton antiporters from different species also enhanced both salt and drought tolerance when being overexpressed in *Arabidopsis* (Brini et al., 2007; Wei et al., 2011). Such dual functions in salt and drought tolerance would allow scientists to efficiently engineer the abiotic stress tolerance of crops, thus leading to improved agricultural production on lands that are marginal due to salt and/or drought problems. Both drought and salt tolerance in soybean were shown to be related to the accumulation of soluble saccharides and proline, and less damage of chlorophyll when stresses occurred (Abd El-Samad and Shaddad, 1997). Such physiological traits and Na^+^, K^+^, and Cl^-^ accumulations in shoots, together with possible alternations of the expressions for genes on ABA signaling pathways, would be excellent targets for the future investigation on the precise physiological and molecular mechanism(s) that drive *GmST1*’s abiotic stress tolerance.

Abiotic stresses usually cause physiological damage to crops and lead to more ROS production that, in turn, damages cell and DNA structures, eventually leading to apoptosis. ROS signaling is known to be involved in regulating plants’ responses to both biotic and abiotic stresses (Mustilli et al., 2002; Jaspers and Kangasjarvi, 2010; Torres, 2010). One way to improve abiotic stress tolerance is to alleviate ROS production during stress periods. In the present study, we discovered that, after a 48-h salt treatment, *GmST1* transgenic lines produced less H_2_O_2_ than that of the wild type control (**Figure 4**). In one way, this result can be interpreted to indicate that salt stress caused less damage to *GmST1* transgenic lines than that to the wild type control. On the other hand, it also implies a possible mechanism that *GmST1* uses in regulating plant abiotic stress tolerance.

In recent studies, Guan et al. (2014b) and Qi et al. (2014) independently identified Glyma03g32900 as a potential candidate gene governing soybean salt tolerance. Our present study demonstrated that *GmST1* conferred strong salt tolerance in *Arabidopsis* transgenic lines. *GmST1* is located on linkage group N and is overlapped with Glyma03g32900. In consistent with these findings, multiple QTL mapping studies for soybean salt tolerance, independently conducted involving different salt-tolerant cultivars and/or different species (wild and cultivated soybeans), also identified a major QTL in this region on linkage group N and suggested that it plays a critical role in improving soybean salt tolerance under different genetic backgrounds (Lee et al., 2004; Hamwieh and Xu, 2008; Tuyen et al., 2010; Hamwieh et al., 2011; Ha et al., 2013; Guan et al., 2014a,b; Qi et al., 2014). The importance of this genomic region in control of soybean salt tolerance warrants a further analysis of genome/gene structures, and molecular and physiological mechanisms of this locus that controls salt tolerance.

In search of salt tolerance candidate genes in soybean, previously a cation/proton antiporter (*GmCAX1*) was identified (Luo et al., 2005). Transgenic *Arabidopsis* lines overexpressing *GmCAX1* accumulated less Na^+^ and were more resistant to salt stress during germination stage (Luo et al., 2005). Additionally, novel sodium/proton antiporters, *GmNHX1*, and *GmNHX2*, were also cloned from soybean (Sun et al., 2006; Zhou et al., 2009). Overexpression of *GmNHX1* in *Lotus corniculatus* also exhibited strong salt tolerance and reduced accumulation of Na^+^ in shoots (Sun et al., 2006), while overexpression of *GmNHX2* in *Arabidopsis* also resulted in salt tolerance at germination and seedling stages (Zhou et al., 2009). *GmST1* was annotated as a cation/proton antiporter. However, the size of *GmST1* is relatively small (Accession KU871394), in comparison with other homologous genes isolated in other plant species (Shi et al., 2000; Brini et al., 2007; Wei et al., 2011). Since cation/proton antiporters belong to transmembrane proteins, the size of *GmST1* does not fit into this group of genes. According to the newly annotated soybean genome database, *GmST1* overlaps with the Glyma.03g171600 gene and belongs to the three prime part of the Glyma.03g171600. In addition, Gm0063x0041, an adjacent (upstream) gene to *GmST1*, was annotated as the five prime part of Glyma.03g171600. However, intron-exon splicing sites predicted in the Glyma.03g171600 model were completely different from those based on the model which interpreted *GmST1* and Gm0063x0041 as individual genes.

Recently Glyma.03g171600 was identified as *GmCHX1* and contributed to soybean salt tolerance (Qi et al., 2014). The intact *GmCHX1* gene was highly expressed in soybean roots, but was barely detected in soybean shoots (Guan et al., 2014b; Qi et al., 2014). Through RT-PCR analysis, we were able to examine if *GmST1* and Gm0063x0041 belong to parts of *GmCHX1* or if they act as individual genes. Using primer combinations of Gm0063x0041 forward (P1 and P2, respectively) and *GmST1* reverse (P6), we failed to amplify any RT-PCR products from either salt-treated or untreated soybean leaves. Though, we could not rule out the *GmCHX1* model given that *GmCHX1* was not expressed in leaf tissues (Guan et al., 2014b; Qi et al., 2014). By examining the expressions of *GmST1* and Gm0063x0041 as individual genes, however, we found that both genes were expressed in soybean leaves. *GmST1* was highly induced by salt treatment in both salt-sensitive and salt-tolerant soybean varieties, but less expressed in the conditions with no salt treatment. On the other hand, Gm0063x0041 was highly expressed in the salt-sensitive soybean Union under both control and salt-treated conditions but only a very low level of expression was detected in the salt-tolerant soybean WF-7. Interestingly, according to the *GmCHX1* model, Gm0063x0041 reverse primer (P3) was located in the intron of *GmCHX1* and should not be able to amplify the RT-PCR product if the *GmCHX1* model was correct, but it did amplify the RT-PCR product. Furthermore, we designed another reverse primer, P5, to further verify the model. In the *GmST1* model, P5 was located in the intron region, but in the *GmCHX1* model, it was located at 3′ UTR. Compared with the P4+P6 combination, the P4+P5 combination did not yield any RT-PCR product at the predicted size. It is clearly indicated that both *GmST1* and Gm0063×00341 are not part of the Glyma.03g171600 gene but two individual genes in soybean leaf tissues, though they are overlapped by the latter. It also implies that the current Glyma.03g171600 gene annotation might need further revision. Based on our data and those reported by Guan et al. (2014b) and Qi et al. (2014), we hypothesize that different intron-exon splicing sites exist in this genomic region. In soybean root tissues, a splicing pattern forms one gene, *GmCHX1*, while in soybean leaf tissues, different splicing patterns create two distinct genes in the same genomic region. This phenomena is an exciting observation and warrants further investigation into the details of intron-exon site reorganization in different organs and genome structures in this genomic region.

## Conclusion

We have cloned *GmST1* from the salt-tolerant soybean variety WF-7, and we have demonstrated that *GmST1* enhances both salt and drought tolerance through *Arabidopsis in planta* study. Furthermore, *GmST1* also increases ABA sensitivity and reduces ROS production upon salt treatment. Additionally, *GmST1* is strongly induced by salt stress in soybean leaf tissues and functions as an intact gene, rather than as a part of the Glyma.03g171600 gene. Further comparative studies between *GmST1* and Glyma.03g171600, as well as on the possible different intron-exon splicing patterns in different organs, will be required to fully describe the real gene structure and gene models within this genomic region. Regardless, the fact that *GmST1* overexpression improved both salt and drought tolerance in *Arabidopsis* suggests that it may be worthwhile to elucidate the contribution of this gene to multi-abiotic tolerance in crops. The overexpression of *GmST1* in crops, including soybean, may provide a new approach to genetically engineering agricultural and economical plants of importance with tolerance to multiple abiotic stresses simultaneously.

## Accession Number

cDNA sequence of GmST1 gene is submitted to GeneBank with accession number KU871394.

## Author Contributions

SR designed experiments, SR, CL, and AP conducted experiments, SR and G-LJ analyzed data and wrote the manuscript.

## Conflict of Interest Statement

The authors declare that the research was conducted in the absence of any commercial or financial relationships that could be construed as a potential conflict of interest.
